# Hypnotism

**Published:** 1889-12-07

**Authors:** 


### HYPNOTISM.

The calm judgment of such an authority as Dr. V. Ziemssen on this practice, which has recently been enthusiastically revived on the Continent, especially in Paris, deserves attention. "Our experience," he said, "of the employment of hypnotism as a therapeutic agent in the Royal Hospital at Munich has been throughout unfavourable. We have carefully observed a large number of cases treated by it, and the results have been in all essential points unsatisfactory, and in a few almost deterrent. I heartily concur in the opinion that hypnotism effects little or no good in slight functional disturbances, and that in many cases it considerably aggravates the conditions. I trust, however, that the sound sense of German physicians, and their scientific objectivity, will oppose a strong barrier to the craving for the marvellous and the unhealthy speculations involved in the whole question of hypnotism."

